# Prevalence and variables associated with depression, anxiety, and stress among Chilean higher education students, post-pandemic

**DOI:** 10.3389/fpsyt.2023.1139946

**Published:** 2023-03-30

**Authors:** Jonathan Martínez-Líbano, Javier Torres-Vallejos, Juan C. Oyanedel, Nicol González-Campusano, Gilda Calderón-Herrera, María-Mercedes Yeomans-Cabrera

**Affiliations:** ^1^Facultad de Educación y Ciencias Sociales, Universidad Andrés Bello, Santiago, Chile; ^2^Facultad de Salud y Ciencias Sociales, Universidad de las Américas, Santiago, Chile

**Keywords:** mental health, depression, anxiety, stress, post-pandemic, prevalence

## Abstract

**Background:**

Mental health among university students is a public health problem in Chile, understanding that this population is susceptible to mental disorders.

**Objective:**

The present study aimed to determine the prevalence and variables influencing depression, anxiety, and stress in Chilean university students.

**Method:**

A representative sample (n = 1,062) of Chilean university students and a cross-sectional study design were used. Bivariate analysis and multiple logistic regression were performed to identify risk factors associated with symptomatology. They were analyzed using descriptive statistics. A questionnaire with sociodemographic variables was applied in November 2022, in addition to the depression anxiety stress scale (DASS-21), instruments with excellent reliability in this population (α = 0.955; ω = 0.956). On the other hand, the Questionnaire of Problematic Alcohol and Drug Consumption (DEP-ADO) was applied. A descriptive analysis was performed, followed by bivariate analysis and multiple logistic regression using SPSS version 25. The variables showed a value of *p* <0.05; they were declared statistically significant in the final model. Odds ratios (OR) were adjusted to a 95% confidence interval (95% CI), which was used to determine the independent predictors.

**Results:**

The prevalence of mental health problems in this population was high, with depressive symptoms in 63.1% of the sample; 69.2% with anxiety; 57% with stress; 27.4% with problematic alcohol consumption; and 14.9% with inappropriate marijuana consumption. Some 10.1% of the sample reported daily medication with antidepressants and/or anxiolytics. Concerning significant variables for depression, these were: being female, belonging to sexual, not having children, having problematic marijuana use, and using prescription drugs. Concerning anxiety, the significant variables were being a woman, belonging to sexual minorities, being an adolescent, and consuming prescription medication. Finally, concerning stress, the significant variables were being a woman, belonging to sexual minorities, being a student dedicated exclusively to academic activities, and taking prescription medication.

**Conclusion:**

Chilean university students presented a high prevalence of anxiety, depression, and stress, where being female and belonging to sexual minorities seem to be the variables that have the greatest impact on susceptibility to mental health problems. These results should call the attention of political and university authorities in Chile to improve this population’s mental health and quality of life since they are the following professional generation of our country.

## 1. Introduction

The World Health Organization defines health as a state of complete physical, mental, and social well-being and not merely the absence of disease or infirmity (1). Similarly, mental health is not only the absence of mental illness, such as anxiety and depression, but also the presence of psychological well-being, such as gratitude, social connectedness, and mindfulness (2).

Mental health problems account for about one-sixth of the global disease burden in young people (3). Approximately 10–20% of the world’s adolescents have experienced mental health problems; moreover, these problems have become the main reasons for developing psychological barriers, such as those leading to risk-taking and even suicide (4). Concerning Chile, an epidemiological study reported that 16.5% had one or more psychiatric disorders during the last 12 months (5). A recent study reported that during the pandemic, 37.1% of college students had elevated symptoms of depression, 37.9% had anxiety symptoms, and 54.6% had symptoms of stress (6).

In contemporary society, depression, anxiety, and stress are much more common than at other times. Recent studies have indicated that young adults suffer from depression and anxiety disorders that affect their emotional and mental well-being (7). These are essential mental health indicators; these symptoms can negatively affect someone if they are not detected early or treated (8).

In Chile, scientific reports in recent decades indicate that depression and anxiety are the disorders with the highest incidence among Chilean university students, with values higher than those found in the general population, both young and adult (9–12). Also, specific problem areas frequently associated with depression and anxiety are self-injurious behavior and suicide, eating disorders, substance abuse, and academic stress (13).

Recent post-pandemic studies revealed that psychiatric symptoms were notably prevalent in the university population. Most participants (67.05%) reported symptoms of post-traumatic stress related to COVID-19; anxiety symptoms were clinically elevated in 34.73% of participants and 46.55% for depression (14).

Based on the above, we hypothesized that depression, anxiety, and stress symptomatology would be elevated after the pandemic. Hence, it is necessary to determine the post-pandemic prevalence of depression, anxiety, and stress, and to determine the sociodemographic variables that affect the incidence of this symptomatology.

## 2. Materials and methods

A cross-sectional quantitative study was conducted in November 2022 on Chilean university students. Sociodemographic questions were entered into Google Forms with the instruments to measure the variables in a descriptive analysis to determine the most significant variables and then perform a bivariate logistic regression to confirm the impact of these variables on the phenomenon being studied in this study. Bivariate logistic regression is one of the most widely used methods around health in general; an attempt is made to model the probability of a dichotomous dependent variable as a function of one or more independent variables (15). This study used a probability sample of higher education students from different institutions and disciplines. The selected sample consisted of 1,062 students—according to data from the Ministry of Education, in 2021, there were 1,204,414 students enrolled in Chilean universities (16). The sample size was associated with a maximum observed error of ±3.3%, assuming maximum variance and a 95% confidence level. Finally, a bivariate analysis and multiple logistic regression were performed to confirm what was identified in the descriptive analysis.

### 2.1. Data collection

The type of sampling was convenience sampling, a non-probabilistic sample selection method used in exploratory research or when random or stratified sampling is not possible (17). Data were collected through a questionnaire hosted on Google Forms and distributed through the social networks that Chilean higher education students preferentially use (18). According to the Helsinki Declaration, all data were treated with confidentiality. Besides, only those questionnaires in which the student signed the informed consent were considered.

### 2.2. Measures/instruments

*The Depression Anxiety Stress Scale (DASS-21)* (19): The DASS-21 scale is composed of three self-report subscales to assess the severity/frequency of anxiety (7 items), depression (7 items), and stress (7 items). Each item is scored from 0 to 3, referring to the previous week of assessment (from “it does not apply to me at all” to “it mainly applies to me”). The depression scale assesses dysphoria, meaninglessness, self-deprecation, lack of interest, and anhedonia. The Anxiety scale considers subjective and somatic symptoms of fear, autonomic activation, situational anxiety, and subjective experience of anxious affect (20). The scale score is calculated by adding the scores of the corresponding items, ranging from 0 to 21 (21), and has an Alpha of 0.85 for the Chilean population (22). For this application, Cronbach’s alpha was α = 0.955, and omega was ω = 0.956. The cut-off points for the depression scale were 5 points, the anxiety scale 5 points, and the stress scale 8 points (20). Degrees of depression are: “no depression” (0–4), “mild depression” (5–6), “moderate depression” (7–10), “severe depression” (11–13), “and extremely severe depression” (≥14). The anxiety grades are “no anxiety” (0–3), “mild anxiety” (4), “moderate anxiety” (5–7), “severe anxiety” (8–9), and “extremely severe anxiety” (≥10). Finally, for stress, the degrees are “no stress” (0–7), “mild stress” (8–9), “moderate stress” (10–12), “severe stress” (13–16), and “extremely severe stress” (≥17).

*Questionnaire for the detection of problematic alcohol and drug use (DEP-ADO)* (23): This is a questionnaire in which the person must give an account of their consumption during the last 12 months. Example: Have you consumed any of these products? If yes, how often have you consumed them? Possible answers range from “I have not consumed” to “I have consumed every day.”

*Questionnaire of sociodemographic variables*: In this section, questions were asked about age, marital status, sex, sexual orientation, children, major, and progress in the academic program, among others (Annex 1).

### 2.3. Statistical analysis

The results were analyzed with the SPSS version 25 statistical package (24). For descriptive statistics, frequency distribution tables and percentages were used. For bivariate analysis, the X2 test was used to determine the differences between the independent variables and the presence of depression, anxiety, and stress. A category analysis was performed using 2 × 2 tables to determine X2 and Odds Ratios in the variables where statistically significant differences were established. Finally, a binary logistic regression was performed to confirm the significant variables in depression, anxiety, and stress.

### 2.4. Ethics statement

This study was developed under the Declaration of Helsinki and was approved by the central bioethics committee of the Universidad Andrés Bello, approval act 024/2022. All participants gave their informed consent expressly in the questionnaire.

## 3. Results

The sample consisted of 1,062 Chilean higher education students aged 18 to 63 years, with a mean of 27.18 years (SD = 8.65). Seventy-two percent of the sample were female, and 28% were male students. 74.6% were students from Chilean universities, and 25.4% were from colleges or technical training institutes. The distribution of the sociodemographic variables and characteristics of the sample can be seen in Table 1.

**Table 1:** Characteristics of the Chilean higher education student sample.

Variables	Category	(*n*)	(%)
Sex	Woman	765	72
	Man	297	28
Age	Adolescent (18–20 years old)	213	20.1
	Young Adult (31–35 years)	673	63.4
	Adult (36–63 years)	176	16.6
Sexual orientation	Heterosexual	902	84.9
	Sexual minority	160	15.1
Couple	With partner	638	60.1
	Without partner	424	39.9
Children	With children	270	25.4
	Without children	792	74.6
City	Large city	654	61.6
	Small city	408	38.4
Nationality	Chilean	1,050	98.9
	Foreign	12	1.1
Study area	Arts and communications	35	3.3
	Basic sciences	36	3.4
	Social sciences	363	34.2
	Education	165	15.5
	Engineering and business	227	21.4
	Health	236	22.2
Activity	Study only	466	43.9
	Work and study	596	56.1
School	University	792	74.6
	Technical institute	270	25.4
Academic progress	First year	332	31.3
	Second year	216	20.3
	Third year	231	21.8
	Fourth year	138	13
	Fifth year	97	9.1
	Graduating	48	4.5

Concerning the prevalence of symptoms of depression, anxiety, and stress in this sample of Chilean university students, we can say that depressive symptoms are present in mild (11.8%), moderate (21.8%), severe (10.3%), and extremely severe (19.2%) forms. Anxiety is present in 6.6% in a mild form, 17.1% in a moderate form, 8.6% in a severe form, and 36.9% in an extremely severe form. Finally, stress is present in 11.5% of cases in a mild form, 17.1% in a moderate form, 17.5% in a severe form, and 10.8% in an extremely severe form (Supplementary Table 1).

When performing the comparative statistical analysis through X2 between the group of students with symptoms of depression, anxiety, and stress with the group that did not present these symptoms, it was determined that the variables statistically associated with the presence of depression were: sex, sexual orientation, age, children, couple relationship, students who only studied and students who studied and worked, sleep disorders, alcohol, and marijuana consumption, and consumption of prescription drugs.

Among the variables significantly associated among students with anxiety symptoms were sex, sexual orientation, age, children, students who only study and students who work and study simultaneously, and consumption of prescription and non-prescription drugs.

Finally, as shown in Table 2, in stress, the associated variables were sex, sexual orientation, children, students who only study and students who work and study simultaneously, and consumption of prescription and non-prescription drugs.

**Table 2:** Analysis of variables with contingency tables to determine statistically significant differences in depression, anxiety, and stress.

Variables	Depression			Anxiety			Stress		
	*X* ^2^	df	*p*-value	*X* ^2^	df	*p*-value	*X* ^2^	df	*p*-value
Sex	8.974	1	0.003	36.622	1	0.000	24.006	1	0.000
Sexual orientation	38.118	1	0.000	30.831	1	0.000	33.833	1	0.000
Age	71.983	43	0.004	82.685	43	0.000	44.430	43	0.411
Children	29.439	1	0.000	16.609	1	0.000	10.534	4	0.001
Relationship	8.960	1	0.003	0.158	1	0.691	0.302	1	0.583
City	0.414	1	0.520	0.317	1	0.574	0.222	1	0.638
Nationality	3.626	1	0.057	2.053	1	0.152	0.720	1	0.396
Exclusively studying	18.798	1	0.000	20.055	1	0.000	12.693	1	0.000
Working and studying	18.798	1	0.000	20.055	1	0.000	12.693	1	0.000
School	0.142	1	0.706	0.461	1	0.497	0.430	1	0.512
Alcohol consumption	3.694	1	0.055	0.255	1	0.614	0.896	1	0.344
Marijuana use	20.375	1	0.000	0.514	1	0.473	0.243	1	0.622
Cocaine use	0.522	1	0.470	1.200	1	0.273	0.214	1	0.644
Use of solvents	0.522	1	0.470	1.200	1	0.273	0.214	1	0.644
Use of hallucinogens	0.522	1	0.470	0.835	1	0.361	0.214	1	0.644
Amphetamine use	1.569	1	0.210	0.179	1	0.672	0.643	1	0.423
Use of non-prescription drugs	1.693	1	0.193	3.878	1	0.049	1.755	1	0.183
Use of prescription drugs	9.979	1	0.002	21.085	1	0.000	18.325	1	0.000

*p < 0.005, **p < 0.001 and 1: constant.

The variables significantly associated were subjected to a category analysis using 2 × 2 tables to determine the odds ratios relative to the categories without symptoms of depression, anxiety, and stress.

For depression, sex, sexual orientation, adolescence, adulthood, exclusive dedication to study, dedication to study and work, sleep difficulties, alcohol use, marijuana use, and prescription drug use were positively associated (Supplementary Table 2).

For anxiety, sex, sexual tendency, having children, adolescence, adulthood, exclusive dedication to their studies, simultaneous dedication to study and work, and consumption of prescription and non-prescription drugs were positively associated (Supplementary Table 3).

As for stress, sex, sexual tendency, having children, exclusive dedication to study, simultaneous dedication to study and work, and consumption of prescription drugs were positively associated (Supplementary Table 4).

From the logistic regression performed with the significant variables is that it is possible to appreciate the predictor variables of depressive symptoms in college students such as being female [AOR = 1.521; 95% CI: 1.116, 2. 074], belonging to sexual minorities [AOR = 0.2.142; 95% CI: 1.495, 3.069], not having children [AOR = 1.661; 95% CI: 1.114, 2.475], problematic marijuana use [AOR = 1.785; 95% CI: 1.222, 2.607], and taking prescription medication [AOR = 1.687; 95% CI: 1.121, 2.541] (Table 3).

**Table 3:** Coefficients, statistical significance, and odds ratio of the predictors of depression studied.

	*B*	S.E.	Wald	df	*p*-value	AOR	95% C.I. for AOR	
							Lower	Upper
Women	0.420	0.158	7.042	1	0.008	1.521	1.116	2.074
Sexual minority	0.762	0.183	17.242	1	0.000	2.142	1.495	3.069
Adolescents	0.222	0.177	1.579	1	0.209	1.249	0.883	1.766
Adults	−0.172	0.234	0.540	1	0.462	0.842	0.532	1.332
No children	0.507	0.204	6.201	1	0.013	1.661	1.114	2.475
Exclusively studying	0.256	0.152	2.819	1	0.093	1.292	0.958	1.741
Alcohol use	0.036	0.158	0.051	1	0.821	1.037	0.760	1.414
Marijuana use	0.579	0.193	8.976	1	0.003	1.785	1.222	2.607
Prescription drug use	0.523	0.209	6.279	1	0.012	1.687	1.121	2.541
Constant	−1.350	0.398	11.498	1	0.001	0.259		

Concerning anxiety, the significant variables were being a woman [AOR = 2.212; 95% CI: 1.644, 2.975], belonging to sexual minorities [AOR = 2.137; 95% CI: 1.476, 3.095], being an adolescent [AOR = 1.568; 95% CI: 1.106, 2.222], and use of prescription drugs [AOR = 2.227; 95% CI: 1.449, 3.422] (Table 4).

**Table 4:** Coefficients, statistical significance, and odds ratios of the anxiety predictors studied.

	*B*	S.E.	Wald	df	Sig.	Exp(B)	95% C.I. for AOR	
							Lower	Upper
Women	0.794	0.151	27.514	1	**0.000**	2.212	1.644	2.975
Sexual minority	0.760	0.189	16.156	1	**0.000**	2.137	1.476	3.095
Adolescents	0.450	0.178	6.373	1	**0.012**	1.568	1.106	2.222
Young adults	−0.381	0.211	3.259	1	0.071	0.683	0.452	1.033
With children	0.158	0.184	0.736	1	0.391	1.171	0.816	1.680
Exclusively studying	0.254	0.148	2.946	1	0.086	1.290	0.965	1.725
Prescription drug use	0.801	0.219	13.338	1	**0.000**	2.227	1.449	3.422
Medications without prescription	0.370	0.502	0.541	1	0.462	1.447	0.541	3.872
Constant	−1.971	0.570	11.975	1	0.001	0.139		

Concerning stress, the significant variables were being a woman [AOR = 2.648; 95% CI: 1.681, 4.172], being part of sexual minorities [AOR = 2.395; 95% CI: 1.613, 3.554], being a student dedicated exclusively to study [AOR = 1.510; 95% CI: 1.064, 2.141], and with consumption of prescription drugs [AOR = 2.123; 95% CI: 1.363, 3.308] (Table 5).

**Table 5:** Coefficients, statistical significance, and odds ratios of the stress predictors studied.

	*B*	S.E.	Wald	df	Sig.	Exp(B)	95% C.I. for AOR	
							Lower	Upper
Woman	0.974	0.232	17.622	1	0.000	2.648	1.681	4.172
Sexual minority	0.873	0.201	18.793	1	0.000	2.395	1.613	3.554
No children	0.393	0.236	2.781	1	0.095	1.482	0.933	2.354
Exclusively studying	0.412	0.178	5.330	1	0.021	1.510	1.064	2.141
Prescription medication consumption	0.753	0.226	11.069	1	0.001	2.123	1.363	3.308
Constant	−0.312	0.264	1.395	1	0.238	0.732		

## 4. Discussion

According to the present study, we can refer that higher education students present high rates of depression (63.1%), anxiety (69.2%), and stress (57%). It can be observed that mental health problems have increased after the COVID-19 pandemic in Chile. In a study conducted in a sample of students in Chile in 2022 with the same instrument and cut-off points, it was determined that the prevalence of depression, anxiety, and stress was already high depression 37.1%, anxiety 37.9%, and stress 54.6% (6). These data match pre-pandemic results—37% anxiety and 38% depression (25). This reinforces our hypothesis that college students’ post-pandemic mental health is at critical levels.

The imposition of long periods of isolation, social distancing, and loss of personal freedoms have produced critical psychological effects, causing mental disorders and emotional distress (26). These have been accentuated in university students given the adaptive academic and personal demands that COVID-19 entails (27, 28), making this population extremely susceptible to mental health problems. From the logistic regression performed, significant variables for depression, anxiety, and stress in university students were identified. The logistic regression model confirms that the sociodemographic variables, women, sexual minorities, not having children, marijuana use, and prescription drug use, were significant for depression (Supplementary Table 5). In the case of anxiety, being a woman, belonging to sexual minorities, being an adolescent, and prescription drug use were significant (Supplementary Table 6). Finally, stress-associated variables were being a woman, belonging to sexual minorities, being exclusively dedicated to their studies, and using prescription drugs (Supplementary Table 7).

### 4.1. On depression and demographic characteristics

As for depression, we can say that it is frequently present among young people (29). However, epidemiological findings associate depression more with women than men (30). Our study determined that being a woman is a risk factor for developing depression more than in men [AOR = 1.521; 95% CI: 1.116, 2.074]. This is consistent with other studies showing that women have been the most affected by the mental health pandemic (31, 32). There is a tendency for women to feel lonelier than men (33), which was accentuated during the pandemic, given the reduction in professional and face-to-face peer support (34). In addition, women are more often victims of domestic violence. During this pandemic, there has been an enormous explosion of this phenomenon because, due to confinement, many people were forced to isolate themselves with their aggressors (1). Likewise, domestic violence has been identified as a predictor of depression (35). Moreover, emotional exhaustion has been higher in women than men during the COVID-19 pandemic (32, 36, 37), which has been directly associated with the increase or onset of depression (38). In addition, the mental health status of women in Latin America depends on several social determinants: unequal access to education and work and their benefits, as well as exposure to violence and lack of adequate reproductive health, which affect their psychological well-being (39–41).

Our study observed that sexual minorities are more likely to present depression [AOR = 0.2.142; 95% CI: 1.495, 3.069]. This can be explained by the fact that, compared to the general population, people belonging to sexual minorities are exposed to higher levels of discrimination and violence, which can lead to physical and psychological problems; specifically, they have a higher prevalence of depression compared to the general population [AOR = 0.2.142; 95% CI: 1.495, 3.069] (42).

On the other hand, students without children are more likely to develop depressive symptoms [AOR = 1.661; 95% CI: 1.114, 2.475]. It should be considered that people without children tend to have less emotional support in critical situations (43). Furthermore, it has been evidenced that married people with children present less burnout than single people (44), and social support has been shown to play a protective role against depression in the general population (45). The results of our study are consistent with others published in the US and Japan, where higher rates of depression were also observed in singles and women (46–50).

### 4.2. On depression and drug use

Marijuana use among young adults has increased considerably recently, even more so among college students (51, 52). Our study associated marijuana use as a possible predictor of the onset of depression [AOR = 1.785; 95% CI: 1.222, 2.607]. Other studies indicate that depression and anxiety appear first, and then, to remedy the emotional distress, marijuana use occurs (53). However, other studies propose the opposite: they hypothesize that marijuana consumption occurs first and, consequently, emotional disturbances arise (54). However, both cases have an association between marijuana use and depression.

On the other hand, taking prescription drugs was a predictor variable for depression [AOR = 1.687; 95% CI: 1.121, 2.541], anxiety [AOR = 2.227; 95% CI: 1.449, 3.422], and stress [AOR = 2.123; 95% CI: 1.363, 3.308]. According to some research, the COVID-19 pandemic caused an increase in the consumption of psychotropic drugs, mainly in women, young adults —between 18 and 39 years of age— and children under 18 years of age (55). The COVID-19 pandemic brought with it an increase in psychological disorders (56, 57), and drug use to cope with the situation of confinement, restrictions, and uncertainty increased considerably (58, 59).

### 4.3. On anxiety and stress, and demographic characteristics

Anxiety and stress among university students are usual, even more so in the prolonged quarantine period they experienced (60). In our study, being a woman appeared as a possible factor in the occurrence of anxiety and stress [AOR = 2.212; 95% CI: 1.644, 2.975]; [AOR = 2.648; 95% CI: 1.681, 4.172]. The COVID-19 pandemic affected women more profoundly than men in several areas, both in the workplace-especially in the health and social sector and at home, with a higher workload due to confinement and quarantine measures (33). This overload in various areas of women’s lives may have increased anxiety and stress.

On the other hand, belonging to sexual minorities appears as a possible risk factor in the appearance of anxiety [AOR = 2.137; 95% CI: 1.476, 3.095] and stress [AOR = 2.395; 95% CI: 1.613, 3.554]. Students who suffer victimization experiences in the university context because of their sexual orientation show higher anxiety levels and lower self-esteem (61). Anxiety can arise when the subject does not know what to expect from the context in which they find themself (62), even more so if this environment is hostile, which is frequent with sexual minorities (63–65). Anxiety generates permanent hyperactivation, leading to parasympathetic system activation. It can appear without an explicit stimulus (66).

In our study, the adolescent group was significantly associated with anxiety [AOR = 1.568; 95% CI: 1.106, 2.222). This may be because this population has higher rates of anxiety disorders than the rest. Adults affected by anxiety disorders often begin their disorders in early adolescence (67). The changes to which adolescents are permanently exposed at this age can increase anxiety (68). This is also in line with access to higher education, which is always a challenge that generates anxiety and distress (69). Added to this is the fear of failure; sometimes, the program they entered may not fit their liking (70, 71).

Along the same lines, stress was significantly associated with exclusive dedication to studies [AOR = 1.510; 95% CI: 1.064, 2.141]. On the one hand, the pandemic was particularly stressful for students in higher education (72, 73). In addition, the move from face-to-face to online classes and the subsequent return to face-to-face classes may have made this period particularly stressful for students (74, 75). In this transition, teaching and learning methods and forms of evaluation also underwent changes, which led students to have adaptation problems (18).

Therefore, we can say that mental health is a general problem that should be addressed from a public health perspective. College students are vulnerable to mental health problems because they experience a double transition: an evolutionary transition from adolescence to adulthood and a vital transition in educational systems (76). As a result, college students are often vulnerable to psychological distress and the onset or aggravation of mental health problems (77).

## 5. Limitations

One of the main limitations is that measuring a phenomenon at a given moment does not allow for generalization. Hence, it is necessary to advance in studies addressing the problem’s evolution. Likewise, the sample was taken through social networks, which implies that the population could not have answered the questionnaire with limited access to the Internet (18).

### 5.1. Future studies

It is necessary to continue studying the possible psychological and social variables that affect the mental health of university students, even more so post-pandemic, where we have seen a dramatic increase in the levels of depression, anxiety, and stress. This population is one of the most sensitive and prone to mental pathologies. In terms of projections, progress needs to be made in improving the understanding of the mental health of women and sexual minorities. Therefore, it is prudent to conduct specific research on these populations to fully understand the phenomenon and advance prevention strategies and effective treatments to improve the quality of life of this population.

## 6. Conclusion

The prevalence of psychological symptoms of depression, anxiety, and stress in Chilean university students was high, as alcohol and marijuana consumption. On the other hand, it was determined that the significant sociodemographic variables of risk for this symptomatology were being female, belonging to sexual minorities, not having children, consuming marijuana, and consuming prescription medication.

For future research, the logistic regression model can be used with the selected variables to predict the risk of depression, anxiety, and stress in higher education students.

These findings should draw the attention of political and university authorities so that effective strategies to curb this second mental health pandemic in the Chilean population are implemented as soon as possible.

## Data availability statement

The original contributions presented in the study are included in the article/Supplementary material, further inquiries can be directed to the corresponding author.

## Ethics statement

The studies involving human participants were reviewed and approved by Central Bioethics Committee of the Universidad Andrés Bello, approval act 024/2022. The patients/participants provided their written informed consent to participate in this study.

## Author contributions

JM-L: conceptualization, methodology, and data curation. JM-L, JT-V, and JO: software and validation. JM-L and M-MY-C: formal analysis, resources, and writing—original draft preparation. NG-C and GC-H: investigation. M-MY-C: writing—review and editing and visualization. JM-L: supervision and project administration. JM-L and M-MY-C: funding acquisition. All authors have read and agreed to the published version of the manuscript.

## Funding

This study has been supported by the following grants: Universidad Andrés Bello, Jorge MIllas Project N DI−02−JM/22; SCIA ANID CIE160009 and FONDECYT 1181533.

## Conflict of interest

The authors declare that the research was conducted in the absence of any commercial or financial relationships that could be construed as a potential conflict of interest.

## Publisher’s note

All claims expressed in this article are solely those of the authors and do not necessarily represent those of their affiliated organizations, or those of the publisher, the editors and the reviewers. Any product that may be evaluated in this article, or claim that may be made by its manufacturer, is not guaranteed or endorsed by the publisher.
