# Supplementary material for: Prevalence and variables associated with depression, anxiety, and stress among Chilean higher education students, post-pandemic
Source: Front Psychiatry. 2023 Mar 30;14:1139946. doi: 10.3389/fpsyt.2023.1139946 (PMC10097937; doi:10.3389/fpsyt.2023.1139946)
Supplement: Supplementary file 1 [file Table_1.DOCX]

**Supplementary Table 1. Degrees of depression, anxiety, and stress in Chilean university students**

|  | Depression | | Anxiety | | Stress | |
| --- | --- | --- | --- | --- | --- | --- |
| Category | n | % | n | % | n | % |
| Normal | 392 | 36.9 | 327 | 30.8 | 457 | 43.0 |
| Mild | 125 | 11.8 | 70 | 6.6 | 122 | 11.5 |
| Moderate | 232 | 21.8 | 182 | 17.1 | 182 | 17.1 |
| Severe | 109 | 10.3 | 91 | 8.6 | 186 | 17.5 |
| Extremely Severe | 204 | 19.2 | 392 | 36.9 | 115 | 10.8 |
| Total | 1.062 | 100 | 1.062 | 100 | 1.062 | 100 |
|  |  |  |  |  |  |  |

**Supplementary Table 2. Odds ratios by category for each variable associated with depressive symptoms.**

| Variables | Category | Depression | | COR (95%CI) |
| --- | --- | --- | --- | --- |
|  |  | **Yes (%)** | **No (%)** |  |
| Sex | Woman | 283 (37%) | 482 (63%) | 1 |
|  | Man | 81 (27.3%) | 216 (72.7%) | 1.56 (1.166, 2.102)* |
| Sexual Orientation | Heterosexual | 275 (30.5%) | 627 (69.5%) | 1 |
|  | Sexual Minority | 89 (55,6%) | 71 (44.4%) | .350 (.248, .493)** |
| Age | Adolescents (18 - 20 years old) | 95 (44,6%) | 118 (55.4) | 1.736 (1.278, 2.358)** |
|  | Young Adult | 232 (34.5%) | 441 (65.5%) | 1.024 (0.787, 1.333) |
|  | Adult | 37 (21%) | 139 (79%) | .455 (0.39, .670)** |
| Children | With Children | 56 (20,7%) | 214 (79.3%) | 1 |
|  | Without Children | 308 (38.9%) | 484 (61.1%) | .411 (0.297, .570)* |
| Dedication | Exclusively Studying | 193 (41,4%) | 273 (58.6%) | 1.757 (1.360, 2.269)** |
|  | Studying and Working | 171 (28,7% | 425 (71.3%) | .569 (0.441, .735)** |
| Sleep Difficulties | With Difficulties | 56 (41.8%) | 78 (58.2%) | 1 |
|  | Without Difficulties | 308 (33.2%) | 620 (66.8%) | 1.445 (0.999, 2.091)* |
| Alcohol Consumption | Problematic Use | 113 (38,8%) | 178 (61,2%) | 1 |
|  | Non-problematic use | 251 (32,6%) | 520 (67,4%) | 1.315 (0.994, 1.740)* |
| Marijuana Use | Problematic Use | 79(50%) | 79(50%) | 1 |
|  | Non-problematic use | 285 (31,5%) | 619 (68,5%) | 2.172 (1.543, 3.057)** |
| Prescription Drug Use | Yes | 55 (47,4%) | 61 (52.6%) | 1 |
|  | No | 309 (32.7%) | 637 (67.3%) | 1.859 (1.260, 2.742)* |
| *p<0.005, **p<0,001 and 1: Constant | | |  |  |

**Supplementary Table 3. Odds ratios by category for each variable associated with anxiety symptoms.**

| Variables | Category | Anxiety | | COR (95%CI) |
| --- | --- | --- | --- | --- |
|  |  | Yes (%) | No (%) |  |
| Sex | Woman | 392 (51.2%) | 373 (48.8%) | 1 |
|  | Male | 91 (30.6%) | 206 (69.4%) | 2.379 (1.790, 3.162)** |
| Sexual Orientation | Straight | 378 (41.9%) | 524 (58.1%) | 1 |
|  | Sexual minority | 105 (65,6%) | 55 (34,4%) | .378 (.266, .537)** |
| Age | Adolescents (18 - 20 years old) | 125 (58.7%) | 88 (41.3%) | 1.948 (.1.436, 2.643)** |
|  | Young adult | 304 (45.2%) | 369 (54.8%) | .967 (.752, 1.242) |
|  | Adult | 54 (30.7%) | 122 (69.3%) | .472 (.333, .667)** |
| Children | With children | 94(34.8) | 176 (65.2%) | 1 |
|  | No children | 389 (49.1%) | 403 (50.9%) | .553 (.415, .737)** |
| Activity | Exclusively studying | 248 (53.2%) | 218 (46.8%) | 1.748 (1.368, 2.233)** |
|  | Studying and working | 235 (39.4%) | 361 (60.6%) | .572 (.448, .731)** |
| Consumption of Non-Prescription Medications | Yes | 14 (66.7%) | 7 (33.3%) | 1 |
|  | No | 469 (45.1%) | 572 (54.9%) | 2.439 (.976, 6.093)* |
| Prescription Medication Consumption | Yes | 76 (65.5%) | 40 (34.5%) | 1 |
|  | No | 407 (43%) | 539 (57%) | 2.516 (1.680, 3.768)** |
| *p<0.005, **p<0,001 and 1: Constant | | |  |  |

**Supplementary Table 4. Odds ratios by category for each variable associated with stress symptoms.**

| Variables | Category | Stress | | COR (95%CI) |
| --- | --- | --- | --- | --- |
|  |  | Yes (%) | No (%) |  |
| Sex | Woman | 162 (21.2%) | 603 (78.8%) | 1 |
|  | Man | 25 (8.4%) | 272 (91.6%) | 2.923 (1.873, 4.560)** |
| Sexual Orientation | Heterosexual | 133 (14.7%) | 769 (85.3%) | 1 |
|  | Sexual Minority | 54 (33.8%) | 106 (66.3%) | .339 (.233, .494)** |
| Children | With Children | 30 (11.1%) | 240 (88.9%) |  |
|  | No Children | 157 (19.8%) | 635 (80.2%) | .506 (.333, .768)* |
| Activity | Exclusively Studying | 104 (22.3%) | 362 (77.7%) | 1.776 (1.292, 2.441)** |
|  | Studying and Working | 83 (13.9%) | 513 (86.1%) | .563 (.410, .774)** |
| Prescription Medication Consumption | Yes | 37 (31.9%) | 79 (68.1%) |  |
|  | No | 150 (15.9%) | 796 (84.1%) | 2.485 (1.621, 3.812)** |
| *p<0.005, **p<0,001 and 1: Constant | | |  |  |

**Supplementary Table 5. Logistic Regression Model for depression.**

|  | B | Error estándar | Wald | gl | Sig. | Exp(B) | 95% C.I. | |
| --- | --- | --- | --- | --- | --- | --- | --- | --- |
|  |  |  |  |  |  |  | Inferior | Superior |
| Mujer | 0.443 | 0.157 | 7.984 | 1 | 0.01 | 1.557 | 1.145 | 2.118 |
| Minoría Sexual | 0.758 | 0.182 | 17.253 | 1 | .000 | 2.134 | 1.492 | 3.052 |
| Sin Hijos | 0.748 | 0.172 | 18.831 | 1 | .000 | 2.114 | 1.507 | 2.963 |
| Consumo Marihuana | 0.616 | 0.183 | 11.312 | 1 | .000 | 1.851 | 1.293 | 2.649 |
| Consumo de Medicamentos Prescritos | 0.498 | 0.207 | 5.774 | 1 | 0.02 | 1.646 | 1.096 | 2.471 |
| Constante | -1.23 | 0.276 | 19.72 | 1 | .000 | 0.294 |  |  |

**Supplementary Table 6. Logistic Regression Model for anxiety.**

|  | | | | | | | | |
| --- | --- | --- | --- | --- | --- | --- | --- | --- |
|  | B | Error estándar | Wald | gl | Sig. | Exp(B) | 95% C.I. | |
|  |  |  |  |  |  |  | Inferior | Superior |
| Mujer | 0.79 | 0.15 | 28.1 | 1 | .000 | 2.209 | 1.648 | 2.962 |
| Minoría Sexual | 0.84 | 0.186 | 20.35 | 1 | .000 | 2.31 | 1.605 | 3.323 |
| Adolescentes | 0.7 | 0.162 | 18.49 | 1 | .000 | 2.009 | 1.462 | 2.76 |
| Consumo de Medicamentos Prescritos | 0.84 | 0.213 | 15.65 | 1 | .000 | 2.322 | 1.529 | 3.524 |
| Constante | -2.05 | 0.29 | 49.92 | 1 | .000 | 0.129 |  |  |

**Supplementary Table 7. Logistic Regression Model for stress.**

|  | | | | | | | | |
| --- | --- | --- | --- | --- | --- | --- | --- | --- |
|  | B | Error estándar | Wald | gl | Sig. | Exp(B) | 95% C.I. | |
|  |  |  |  |  |  |  | Inferior | Superior |
| Mujer | 0.945 | 0.231 | 16.68 | 1 | .000 | 2.573 | 1.635 | 4.049 |
| Minoría Sexual | 0.948 | 0.197 | 23.1 | 1 | .000 | 2.579 | 1.753 | 3.796 |
| Solo Estudia | 0.521 | 0.168 | 9.647 | 1 | .000 | 1.683 | 1.212 | 2.339 |
| Consumo de Medicamentos Prescritos | 0.752 | 0.226 | 11.09 | 1 | .000 | 2.121 | 1.363 | 3.301 |
| Constante | -0.86 | 0.351 | 5.969 | 1 | 0.02 | 0.424 |  |  |
|  | | | | | | | | |
