# Supplementary material for: Prevalence and variables associated with depression, anxiety, and stress among Chilean higher education students, post-pandemic
Source: Front Psychiatry. 2023 Mar 30;14:1139946. doi: 10.3389/fpsyt.2023.1139946 (PMC10097937; doi:10.3389/fpsyt.2023.1139946)
Supplement: Supplementary file 2 [file Table_2.DOCX]

| **SOCIODEMOGRAPHIC VARIABLES QUESTIONNAIRE**  **FOR CHILEAN HIGHER EDUCATION STUDENTS** | |
| --- | --- |
| **SEX** | **MARITAL STATUS** |
| ______ Female | ______ Single |
| ______ Male | ______ Married. |
| **NATIONALITY** | ______ Widowed. |
| ______Chilean | ______ Divorced. |
| ______Foreigner | ______ Civil partner |
| **SEXUAL TENDENCY** | **CHILDREN** |
| ______Heterosexual | ______ No children |
| ______Homosexual | ______ Yes, 1 |
| ______Bisexual | ______ Yes, 2 |
| ______Other | ______ Yes, 3 |
|  | ______ Yes, 4 or More |
| **AGE** | **CURRENT AVERAGE GRADE (1 TO 7)** |
| ____________________________ | ______________________________ |
| **MAJOR** | **NUMBER OF FAILED COURSES** |
| **_____________________________** | ______________________________ |
|  |  |
| **GEOGRAPHICAL REGION** | **TYPE OF SCHOOL** |
| ______ Arica and Parinacota Region | ______Technical Training Center |
| ______ Tarapacá Region | ______Professional Institute |
| ______ Antofagasta Region | ______University/College |
| ______ Atacama Region |  |
| ______ Coquimbo Region |  |
| ______ Valparaíso Region |  |
| ______ Metropolitan Region |  |
| ______ O'Higgins Region |  |
| ______ Maule Region |  |
| ______ Ñuble Region |  |
| ______ Biobío Region |  |
| ______ Araucanía Region |  |
| ______ Los Ríos Region |  |
| ______ Los Lagos Region |  |
| ______ Aysén Region |  |
| ______ Magallanes Region |  |
| ______ Biobío Region |  |
| ______ Araucanía Region |  |
| ______ Los Ríos Region |  |
| ______ Los Lagos Region |  |
| ______ Aysén Region |  |
| ______ Magallanes Region |  |
|  | |
